# Anthelmintic efficacy against gastrointestinal nematodes in goats raised under mountain farming conditions in northern Italy

**DOI:** 10.1186/s12917-019-1968-8

**Published:** 2019-06-27

**Authors:** C. Lambertz, I. Poulopoulou, K. Wuthijaree, M. Gauly

**Affiliations:** Faculty of Science and Technology, Free University of Bozen-Bolzano, Universitätsplatz 5, 39100 Bolzano, Italy

**Keywords:** Anthelmintic resistance, Goat, Mountain farms, Endoparasites, Northern Italy

## Abstract

**Background:**

This study aimed to evaluate the efficacy of anthelmintics in goats raised under mountain farming conditions in northern Italy. On 8 goat farms (*n* = 143 animals), a faecal egg count reduction (FECR) test was done after farmers conducted their routine anthelmintic treatments. Furthermore, on 5 goat farms (*n* = 135 animals) a FECR test was done under controlled conditions applying oral formulations of a macrocyclic lactone (ML), benzimidazole (BZ) (partly in combination with salicylanilide (SA)) or a combination of imidazothiazole (IT) and SA on the same farm. AR was assumed if FECR and the upper confidence interval (CI) was < 95% and the lower 95% CI was < 90%.

**Results:**

Underdosing was found in 6 of the 8 farms tested after routine treatments. Out of the 6 routinely ML-treated goat flocks, only three were found where ML showed adequate efficacy. FECR in all others ranged between 64 and 93%. In one flock *Trichostrongylus* spp. and in one *Haemonchus* spp. larvae were identified after treatment. BZ-treated flocks had an efficacy of 99 and 37%. Larvae identified after treatment were *Trichostrongylus* spp. in one and *Haemonchus* spp. in the other flock. Under controlled conditions, ML had an adequate efficacy on 4 farms and a FECR of 88% on another one. BZ was effective on all farms. The combination of BZ and SA had a FECR of 99% on the farm it was tested. IT + SA in combination was effective on 2 farms and had a FECR of 91% on a third farm. Larvae identified after treatment were composed of *Haemonchus* spp. (ML and BZ), *Trichostrongylus* spp. (BZ) and *Teladorsagia* spp. (BZ and SA).

**Conclusions:**

This first report on the prevalence of AR in goats in the mountainous region of South Tyrol reveals a low efficacy of the most commonly used anthelmintics after routine treatments. This might be explained by a high level of underdosing as observed in the farms. However, results from the controlled FECR tests suggest that the observed level of AR was lower but cannot be solely explained by underdosing.

## Background

Given the great economic losses endoparasites cause in small ruminants because of reduced weight gains, decreased milk yields, discarded organs at slaughter and even deaths, regular whole-flock treatments with anthelmintics is still the most commonly used measure to control endoparasitic infections in small ruminants [1, 2]. However, their decreasing efficacy as a consequence of its regular use has gained interest and anthelmintic resistances (AR), especially of gastrointestinal nematodes, in goats were for example already proven in Norway and France [3, 4]. Recently, an alarming number of dairy goat flocks in Northern Italy was found with problems of AR, which emphasized the need for strategies to prevent AR development [5].

Under mountain farming conditions the commonly practiced alteration between pasture areas at lower altitudes in spring and autumn and communal summer pastures at high altitudes in summer beside with and a barn period without access to pasture in winter might impact parasitological infections and the development of AR. The implementation of control strategies is even more complicated compared to larger-scale production conditions, where the adoption of alternative GIN control measures such as the management of refugia, rotation of pasture areas or targeted (selective) treatments by farmers is already low [6]. This holds especially true for many alpine regions such as South Tyrol, Northern Italy where small ruminants constitute an important proportion of the livestock population but are predominantly raised by small-scale or hobby farms. Recently, more than 100 small ruminant farmers were surveyed in this region and it was reported that farmers perceive gastrointestinal nematodes as the most frequent parasites with more than 90% of the farmers applying anthelmintic treatments at least once per year with very limited alteration of anthelmintics [7]. Also, there was almost no use of coprological examinations to validate efficacy, even though 18% of the goat farmers already perceived that anthelmintics were not or only partly effective.

Therefore, the efficacy of several anthelmintics in goats raised under mountain farming conditions in northern Italy was assessed after routine treatments of farmers and under controlled conditions in this study.

## Results

### Faecal egg count reduction (FECR) test of routine anthelmintic treatments

The average flock size of the 8 farms that were included in the FECR test of routine anthelmintic treatments was 29 (range 8–63). Four goat farms raised the animals for milk production, the others for meat production. Boer goats (1 flock), German fawn (1 flock), Saanen (2 flocks), and Passeirer mountain goats (4 flocks) were kept. Two farms were certified as organic farms. Only 2 farms were managed in full-time, the others in part-time. Animals of 4 goat farms that did not practice milking had access to pasture at least from April until October and grazed on communal pasture areas at altitudes above 1500 m during summer months. Four goat farmers perceived GIN to occur regularly on their farm. Whole flock treatments were done by 3 farms once and 3 farms twice per year. Both other farms practiced selective treatments after coprological analysis. Four farmers perceived previous treatments not to be effective. In these treatments, eprinomectin, albendazole and oxfendazole were administered at the dose recommended for sheep and moxidectin at 2 times the sheep label dose. None of the farmers used coprological analysis prior to treatment, but two used it after treatment for efficacy control.

For the treatments assessed in this study, MLs were applied on 6 and BZs on 2 farms. Of the ML-farms, 1 each applied Ivomec® (ivermectin, Merial Italia S.p.a., subcutaneously), Closivet® (ivermectin, Bayer S.p.a., subcutaneously), Cydectin® (moxidectine, Pfizer Italia S.p.a., subcutaneously), Eprinex® (eprinomectin, Merial Italia S.p.a., pour-on) and 2 Oramec® (ivermectin, Merial Italia S.p.a., orally). While the two-fold sheep label dosage was used in 1 farm applying Cydectin® and 1 using Ivomec®, the 4 other farms applying a ML used the sheep label dose only. For the BZ-farms, Zodalben® (albendazole, Calier Italia S.p.a., orally) was used at the sheep label dose (Table 1). *Haemonchus* spp. and *Trichostrongylus* spp. were the most prevalent genera as revealed by larval cultures. Out of the 6 ML-treated flocks, only 3 were found with an adequate efficacy. FECR in all others ranged between 64 and 93%, while median FECs were greater than 0 in only 2 farms. In 1 flock *Trichostrongylus* spp. and in one *Haemonchus* spp. larvae were identified after treatment, while cultures did not yield larvae from the other farms. Low anthelmintic efficacy was also found in one of the two BZ-treated flocks with an efficacy of 37%. Larvae identified after treatment were *Trichostrongylus* spp. in one and *Haemonchus* spp. in the other flock.

**Table 1:** Applied anthelmintics, mean, median and range of faecal egg counts (FEC) before and 10 to 14 days after treatment, arithmetic mean and 95% confidence interval of faecal egg count reduction (FECR, %) and larval identification (LI, %) before and after routine anthelmintic treatments of 8 goat flocks

Farm	*n*	Anthelmintic (class)	pre-FEC			post-FEC			FECR mean	95% CI	LI (pre−/post-treatment)			
			Mean	Median	Range	Mean	Median	Range			Tr	Te	Ha	Other
1	13	Ivermectin (ML)*	729	802	50–1422	4	0	0–602	99	97–100	39/-	0/-	61/-	0/-
2	19	Ivermectin (ML)**	814	600	150–2129	0	0	0–597	99	99–100	59/-	0/-	41/-	0/-
3	30	Eprinomectin (ML)	3462	2636	50–19714	231	351	0–2743	93	85–99	86/100	0/0	14/0	0/0
4	8	Ivermectin (ML)***	953	472	50–2874	11	0	0–200	99	94–100	7/-	0/-	93/-	0/-
5	17	Ivermectin (ML)	211	199	57–622	76	99	0–530	64	41–87	0/0	0/0	100/100	0/0
6	10	Moxidectin (ML)**	704	377	86–2167	2	0	0–417	88	66–100	83/-	0/-	17/-	0/-
7	27	Fenbendazole (BZ)***	1177	955	251–3168	2	0	0–597	99	99–100	25/0	13/0	62/100	0/0
8	19	Fenbendazole (BZ)	1347	900	200–3750	854	616	296–1463	37	19–53	80/100	0/0	20/0	0/0

*n* number of animals, *Tr Trichostrongylus*, *Te Teladorsagia*, *Ha Haemonchus*, Other include *Cooperia*, *Bunostomum* and *Oesopaghostomum*, *: of the ML-farms, 1 each applied Ivomec® (ivermectin, Merial Italia S.p.a., subcutaneously), Closivet® (ivermectin, Bayer S.p.a., subcutaneously), Cydectin® (moxidectine, Pfizer Italia S.p.a., subcutaneously), Eprinex® (eprinomectin, Merial Italia S.p.a., pour-on), 2 farms applied Oramec® (ivermectin, Merial Italia S.p.a., orally), in the BZ-farms, Zodalben® (albendazole, Calier Italia S.p.a., orally) was used at the sheep label dose; −: no larvae in culture; ** administered at 2 times the label sheep dose; *** organic farming

### Faecal egg count reduction (FECR) test under controlled anthelmintic treatments

The average flock size was 26 (range 16 to 37). Animals on all farms were raised for meat production and grazed on different pasture areas during summer months. Farmers raised Passeirer mountain goats (4 flocks) and German fawn (1 flock). One farm was certified as organic farm. Except for 1 farm, all farms were managed in part-time. All animals had access to pasture at least from April until October and grazed on different communal pasture areas at altitudes above 1500 m during summer months. Three goat farms practiced whole-flock treatments once and two twice annually. Two farms perceived previous treatments as not effective, whereas neither of the farmers used coprological analysis for efficacy control.

Mean pre-treatment FECs excluding animals with zero egg count varied between farms and within the farms between groups from 170 to 985 (Table 2). The following products were applied at the two-fold dosage (1.5-fold for the product containing levamisole) than recommended for sheep, ivermectin (ML, Oramec®, 0.8 mg/ml, 0.5 ml/kg bodyweight; Merial Italia S.p.a.), netobimin (BZ, Hapadex®, 50 mg/ml, 0.3 ml/kg bodyweight; MSD Animal Health S.r.l.), fenbendazole (BZ, Panacur®, 25 mg/ml, 0.4 ml/kg bodyweight; MSD Animal Health S.r.l.), a combination of oxfendazole (BZ) and closantel (SA) (Oxydrench®, 25 mg/ml oxfendazole, 50 mg/ml closantel, 0.4 ml/kg bodyweight; Bayer S.p.A) and a combination of levamisole (IT) and oxyclozanide (SA) (Toloxan®, 12.73 mg/ml levamisole, 30 mg/ml oxyclozanide, 0.6 ml/ kg bodyweight; Azienda Terapeutica Veterinaria S.r.l). From the total of 16 groups studied on the 5 farms, mean post-treatment FEC were different from zero in 11 groups and ranged up to 46. Median post-treatment FEC ranged between 23 and 43 in 3 of the groups. Ivermectin (ML) was effective on 4 farms and reached a FECR of 88% on the other farm. Netobimin (BZ) was effective on all the 4 farms it was tested. Fenbendazole (BZ) reached a FECR of 96 to 99% on the 3 farms it was applied. The combination of oxfendazole (BZ) and closantel (SA) was applied on one farm only and had a FECR of 99%. Levamisole (IT) + oxyclozanide (SA) was effective on 2 farms and had a FECR of 91% on the third farm.

**Table 2:** Results of the faecal egg count reduction (FECR, %) test before and 14 days after controlled anthelmintic treatments of 5 goat flocks

Farm	n	Anthelmintic (class)	pre-FEC			post-FEC			FECR mean	95% CI
			Mean	Median	Range	Mean	Median	Range		
1	13	Ivermectin (ML)	713	698	93–1771	11	0	0–471	99	94–100
	6	Netobimin (BZ)	985	384	114–3296	46	23	0–904	95	84–100
	11	Oxfendazole (BZ) + closantel (SA)	393	253	50–1130	3	0	0–448	99	96–100
	7	Levamisole (IT) + oxyclozanide (SA)	535	413	166–828	12	0	0–302	98	91–100
2	6	Ivermectin (ML)	867	326	84–2791	0	0	0	100	99–100
	4	Netobimin (BZ)	588	237	147–1050	0	0	0	100	99–100
	6	Levamisole (IT) + oxyclozanide (SA)	671	384	110–3093	2	23	0–893	91	66–100
3	9	Ivermectin (ML)	410	400	50–900	0	0	0	100	99–100
	6	Netobimin (BZ)	516	461	316–694	0	0	0	100	99–100
	12	Fenbendazole (BZ)	368	262	78–842	5	0	0–54	99	99–100
4	4	Ivermectin (ML)	197	134	55–225	24	43	0–106	88	61–100
	11	Fenbendazole (BZ)	170	125	52–303	1	0	0–68	99	97–100
	5	Levamisole (IT) + oxyclozanide (SA)	189	174	99–409	0	0	0	100	99–100
5	14	Ivermectin (ML)	595	306	115–2253	1	0	0–733	99	99–100
	8	Netobimin (BZ)	818	439	60–2268	9	0	0–199	99	95–100
	3	Fenbendazole (BZ)	476	359	128–473	21	0	0–50	96	80–100

*n* number of animals

*Trichostrongylus* spp. (41%), *Teladorsagia* spp. (24%) and *Haemonchus* spp. (35%) larvae were identified before treatments. Larvae identified in post-treatment samples were composed of *Haemonchus* spp. (ML and BZ), *Trichostrongylus* spp. (BZ) and *Teladorsagia* spp. (BZ and SA) (Table 3). AR to multiple anthelmintics was found on neither of the farms.

**Table 3:** Results of the larval identification (LI; %) before and 14 days after controlled anthelmintic treatments of 5 goat flocks

Anthelmintic (class)	*n*	post-treatment LI			
		Tr	Te	Ha	Other
Ivermectin (ML)	46	0	0	100	0
Netobimin (BZ)	24	69	0	31	0
Fenbendazole (BZ)	26	100	0	0	0
Oxfendazole (BZ) + closantel (SA)	11	0	100	0	0
Levamisole (IT) + oxyclozanide (SA)	18	0	100	0	0

*n* number of animals, *Tr Trichostrongylus*, *Te Teladorsagia*, *Ha Haemonchus*, Other include *Cooperia*, *Bunostomum* and *Oesopaghostomum*

The risk for an insufficient anthelmintic efficacy, i.e. FECR < 95%, was assessed by calculating odds ratios for age classes and BCS. None of these effects was significant at *P* < 0.05, so that odds ratios are not presented in more detail.

## Discussion

The aim of this study was to evaluate the occurrence of AR after routine anthelmintic treatments with commonly used drug formulations in goats (8 flocks) and after controlled treatments applying different anthelmintics on the same farm (5 flocks). The study was conducted under the specific conditions of mountain farming. Alternatives to the use of veterinary drugs such as selective treatments in order to reduce the risk of the development of AR are far from being adopted in many countries [8, 9]. For the studied region this was pointed out by a recent survey and prevalence study involving more than 120 sheep and goat flocks [7]. One major reason why the implementation of AR-limiting strategies is complicated under mountain conditions is that animals from various farms are usually grazing together on communal grazing land without regulations on the use of anthelmintic treatments for these animals being in place. If treatments are conducted, commonly whole-flock treatments once or twice annually with a very limited rotation of applied anthelmintics and without faecal sampling for efficacy control are done [7]. From a farmer’s point of view, whole-flock treatments are warranted, because animal care and treatments during the summer grazing period at high altitudes are further complicated. Also, farms which are generally small-scale, are most commonly run as part-time or hobby farms with very limited income generated by small ruminants, so that veterinary care is limited, too.

AR to a wide range of GIN in goats are proven in a growing number of countries, such as England and Wales [10], France [3], Norway [4], Germany [11], Austria [12] and Switzerland [13]. In central Italy, an alarming number of dairy goat flocks in northern Italy with problems of AR clearly emphasize the need for strategies to prevent AR development in Italy [5].

Results of the present study confirm studies that proved a wide occurrence of AR in small ruminants in many European countries. Even though studies under conditions of mountain farming are very limited, the risk for AR has to be considered high due to the specific factors mentioned above. First it has to be mentioned that only 2 out of the 8 farms used the two-fold sheep label dosage (1.5-fold for products containing levamisole), which clearly supports previous reports on the incorrect dosage used by veterinarians and farmers [14]. The application of the correct dosage is consequently a first and easy-to-implement way to reduce the risk for the development of AR in goats. Referring to the small-scale structure of the farms where farmers generally not intensively address the problem of AR development, veterinarians are the key persons to give advice on the correct drug use. Also, veterinarians should consider altering anthelmintic classes after farmers have experienced inadequate efficacy of previous treatments and especially after AR has been detected. The available studies on the prevalence of AR usually conducted treatments under controlled conditions, so that comparable results under routine conditions as assessed in this study are not available to the author’s knowledge, yet. Consequently, factors such as an incorrect dosage because of wrong estimations of animal weights to calculate the dosage or using the same dosage for sheep and goats, which may be prominent under practical, especially small-scale farming conditions, are not considered.

The study design to test AR under controlled conditions applying different oral formulations on the same farm is generally compliant with the recommendations made by the WAAVP [15]. Post-treatment sampling is proposed at 14 days after treatment when multiple drugs are tested. This was recently suggested even for BZ treatments [16]. Given the fact that farms in the studied region are of small-scale, the number of animals sampled per farm was sometimes low. Also, animals to be included in the study were not selected for high individual pre-treatment FEC values and treatment groups were not blocked by pre-treatment FEC. Nevertheless, valid conclusions can be drawn for the various applied anthelmintics from the total number of animals with data on FECR and given the observed pre-treatment FEC levels. The low number of farms, which were not selected based on the potential risk for AR, nevertheless warrant further studies to validate findings. Arithmetic means were used instead of geometric ones to avoid bias in calculating efficacy [17].

Results of the treatments conducted under controlled conditions clearly support findings following the routine treatments, though the prevalence of inadequate anthelminthic efficacy was lower. AR against ML and IT + SA was found on 1 farm each, with *Trichostrongylus* spp., *Teladorsagia* spp. and *Haemonchus* spp. being found in post-treatment larval cultures. Farms with AR to multiple drugs were not found, contrary to recent findings in dairy goats from a neighboring region of Northern Italy [5].

As anthelmintic treatments will remain the predominant measure to control GIN infections in small ruminants in the near future, targeted selective treatments to maintain refugia of susceptible GIN should be considered by farmers and veterinarians alike, given the inadequate efficacy proven in this and many other regions. The use of coprological analysis to identify individual animals which need to be treated, however, must be considered as a major challenge, especially under the specific conditions of mountain farming. Decisions for anthelmintic treatment based on body condition are too vague and need to be combined with pooled faeces sampling [18, 19]. This may be a feasible starting point for a wide range of farmers and veterinarians, even under extensive production conditions with limited financial resources for animal treatments.

## Conclusions

This first report on the prevalence of AR in goats in the mountainous region of South Tyrol reveals a low efficacy of the most commonly used anthelmintics after routine treatments in the region. This may largely be explained by underdosing, because AR was only found in 2 out of 16 treatment groups following controlled treatments. Though the implementation of alternative measures to control GIN infections under mountain farming conditions may be further complicated, the inadequate efficacy of treatments demand immediate actions. As a first step, a correct application, i.e. dosage, and alteration of available anthelmintic classes after farmers have experienced inadequate efficacy of previous treatments and especially after detection of AR has to be ensured. Because anthelmintic treatments will remain the predominant measure to control GIN infections in the near future, targeted selective treatments may be a step to reduce the risk of AR-development.

## Methods

The applied sampling protocols met the International Guiding Principles for Biomedical Research Involving Animals as issued by the Council for Laboratory Animal Science (ICLAS).

### Selection of farms

Details on the selection of farms are described by Lambertz et al. (2018) [7]. In brief, the study was conducted in the province South Tyrol, northern Italy (46.73° North, 11.29° East). Goat farmers were invited to participate in the study with a letter describing the purpose of the study, providing assurance of confidentiality, asking for permission to publish the anonymous responses and the willingness to participate in the evaluation of the anthelmintic efficacy. A questionnaire with 32 closed and 3 open questions was used to collect data on farm management, system, herd size, breeds, other livestock on the farm, sizes, elevations and management of pastures, drenching practices including the choice of anthelmintics, application practices, rotation of anthelmintics and the perceived effectiveness and side effects. From this dataset of 55 goat flocks, 8 farmers agreed to participate in a faecal egg count reduction (FECR) test after conducting their routine anthelmintic treatments. Furthermore, 5 farmers agreed to participate in a FECR test under controlled conditions. On these 5 farms 3 different anthelmintics were used, a macrocyclic lactone (ML), benzimidazole (BZ) (partly in combination with salicylanilide (SA)) or a combination of imidazothiazole (IT) and SA were applied. The recommendations by the World Association for the Advancement of the Veterinary Parasitology (WAAVP) regarding the detection of AR in nematodes were followed [15]. FECR test was performed once per farm either in autumn 2015 or spring 2016.

### Faecal egg count reduction (FECR) test of routine anthelmintic treatments

Farmers applied their routine treatments against gastrointestinal nematodes using commercial anthelmintics selected by their attending veterinarians following the manufacturers’ instructions for dosage. As a pre-condition for participating in this study, the animals were not treated with anthelmintics within the previous 3 months. The entire flocks were treated, and all animals were naturally infected with GIN. Farmers and attending veterinarians were not advised to select specific anthelmintics. Individual feces samples were taken prior and 10 to 14 days after anthelmintic application. Only individuals older than 3 months were sampled.

### Faecal egg count reduction (FECR) test under controlled anthelmintic treatments

Prior to the application of anthelmintics, animals were weighed individually to calculate the correct dosage. Animals were randomly allocated to one of three groups at each farm. These were treated with oral formulations of either a ML, BZ (partly in combination with SA) or a combination of IT and SA. The following products were applied at the two-fold dosage (1.5-fold for the product containing levamisole) than recommended for sheep, ivermectin (ML, Oramec®, 0.8 mg/ml, 0.5 ml/kg bodyweight; Merial Italia S.p.a.), netobimin (BZ, Hapadex®, 50 mg/ml, 0.3 ml/kg bodyweight; MSD Animal Health S.r.l.), fenbendazole (BZ, Panacur®, 25 mg/ml, 0.4 ml/kg bodyweight; MSD Animal Health S.r.l.), a combination of oxfendazole (BZ) and closantel (SA) (Oxydrench®, 25 mg/ml oxfendazole, 50 mg/ml closantel, 0.4 ml/kg bodyweight; Bayer S.p.A) and a combination of levamisole (IT) and oxyclozanide (SA) (Toloxan®, 12.73 mg/ml levamisole, 30 mg/ml oxyclozanide, 0.6 ml/ kg bodyweight; Azienda Terapeutica Veterinaria S.r.l). Individual faeces samples were taken prior and 14 days after anthelmintic application. The age of the animals was recorded during faeces sampling and body condition score (BCS) was recorded on a 1 to 5 scale [20]. Treatments were conducted during the barn period in autumn 2016.

### Parasitological measurements

Fresh faecal samples were directly collected from the rectum of the individual animals. Samples were stored cool (4 °C) until analysis. Samples prior and after routine treatments to calculate faecal egg count reduction (FECR) were available from a total of 143 goats. For the controlled treatments FECR were calculated for a total of 135 goats. A modified McMaster method was applied for faecal egg counts (FEC). As flotation fluid 60 ml of saturated NaCl solution (specific gravity = 1.2) and 4 g of feces were used [20]. FECR was calculated using the following formula, in which each host served as its own control:

$$ \mathrm{FECR}=\left(1/n\right)\Sigma \Big(100\mathrm{x}\left(1-\left(\left[{T}_{i2}-{T}_{i1}\right]\right)\right) $$

where *T*_*i*2_ is post-treatment and *T*_*i*1_ is pre-treatment FEC in host *I* from a total of *n* hosts [19]. Animals with a zero egg count (< 50 eggs per gram) at the first sampling were excluded from the calculation of FECR. AR was assumed if FECR and the upper confidence interval (CI) was < 95% and the lower 95% CI was < 90% [15]. When neither of the two criteria were met, AR was considered suspected.

For further identification of nematode species, third-stage larvae (L3) were identified according to the Baermann technique [21]. Pooled feces (10 to 20 g) were cultured. For farms that participated in routine treatments, one pooled sample from all animals of one specific flock was used. Under controlled anthelmintic treatments one sample was prepared per treatment group. The first 100 randomly selected L3 of each sample were identified as *Teladorsagia* spp.*, Trichostrongylus* spp.*, Oesophagostomum* spp.*, Chabertia* spp.*, Haemonchus* spp.*, Bunostomum* spp. and *Cooperia* spp. by microscopy [22]. In case fewer than 100 L3 were isolated from a sample, the percentage of larval type was calculated based on the counted L3.

### Statistical analysis

The statistical analysis was performed with the “eggCounts”-package of the R-package [23, 24], according to the WAAVP [15]. Results of the routine treatments and controlled treatments were analysed separately. The paired model was used to calculate FECR and 95% CI using individual FECs before and after treatments for each single farm.

In order to estimate the risk of AR, a logistic regression analysis was performed with the GLIMMIX procedure of the SAS statistical package version 9.3 (SAS Institute., Cary, NC, 2010) for the flocks that were treated under controlled conditions. Individual FECR was transformed into a binary variable where class 0 represented an adequate FECR of > 95% and class 1 a FECR of < 95%, and thus the risk of an insufficient anthelmintic efficacy. The effect of pre-treatment FEC, applied anthelmintic, BCS and age classes were tested. The following age classes were used: < 6 months, 6–12 months, 1–2 years, 2–3 years, 3–4 years and > 4 years. Farm was included as random effect and results are presented as odds ratio and 95% CI. Significance was accepted for *P* < 0.05.

## Data Availability

The datasets generated and analysed during the current study are not publicly available but are available from the corresponding author on reasonable request.

## Abbreviations

AR: Anthelmintic resistance
BCS: Body condition score
BZ: Benzimidazole
CI: Confidence interval
FECR: Faecal egg count reduction
GIN: Gastrointestinal nematodes
IT: Imidazothiazole
ML: Macrocyclic lactones
SA: Sanicylanilide
